# Sea change in coastal science

**DOI:** 10.1038/s41467-020-18333-8

**Published:** 2020-09-15

## Abstract

Coastal regions are disproportionately affected by the impacts of climate change. Preserving the ecological, economic and societal benefits of these environments will rely on synergy across disciplines.

Even though coasts make up just 10% of Earth’s land area, nearly half of the global population lives within 100 km of the sea. Drawn by the advantages afforded by resources, employment opportunities, and recreational activities, the number of people settling on the coasts has increased over the past 50 years at a rate faster than inland regions. But such increases in coastal populations have come with pollution, damaging land use changes, and overfishing that alter ecosystems and diminish the appealing lifestyle and livelihood afforded by coastal living. In addition to the direct impacts caused by coastal habitation, coasts are at risk from rising sea levels and extreme weather events driven by anthropogenic climate change. A new collection from Nature Communications spans coastal ecology, biogeochemistry, and physical processes. These works underscore the unique features of the coastal environment which set it apart from purely terrestrial or marine realms. Together, they highlight that to surmount the challenges faced by Earth’s coasts, coastal science must be considered a unique discipline in its own right.
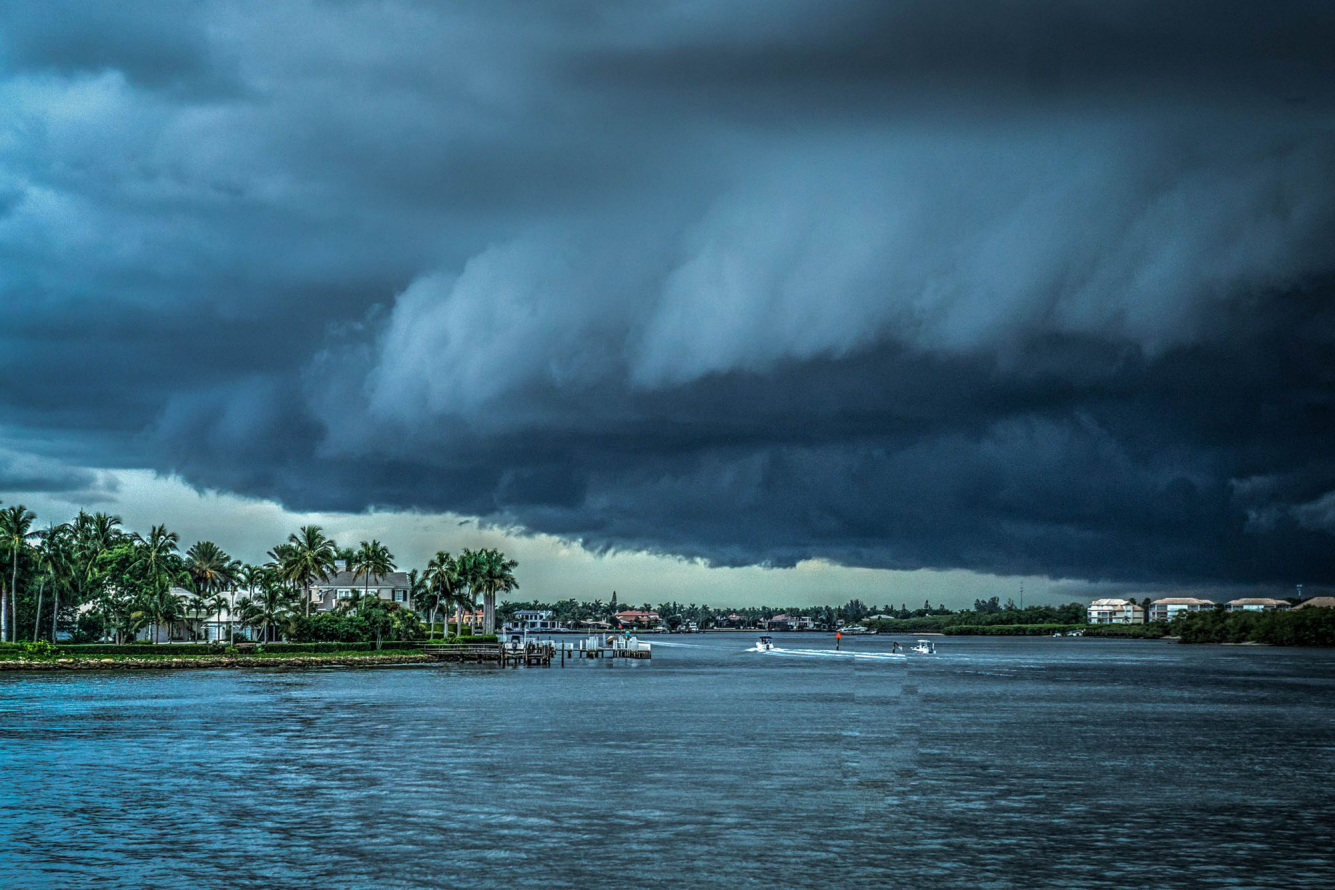


“To surmount the challenges faced by Earth’s coasts, coastal science must be considered a unique discipline in its own right.”

Rising seas attributed to anthropogenic forcing are the canonical climate change impact on coastal systems. Over the past century, the rate of global mean sea level rise has accelerated, doubling to a value of nearly 4 millimetres per year for the past decade^1^. On a global scale, a yearly increase of a few millimetres may seem trivial, but these impacts are not evenly distributed. Island nations like Tuvalu and the Marshall Islands in the South Pacific, and low lying regions of the Chesapeake Bay in the United States are already losing land and forcing relocation of residents. With sea level elevations projected to reach up to a metre by the year 2100 under the worst case emissions scenarios^1^, threatened coastal communities will need to adapt by building seawalls or other protective systems, or risk being swallowed by the sea. Migration and construction of infrastructure are only superficial solutions to a complex web of problems that are only considered in isolation—changing coastal conditions will continue to negatively impact nearshore ecosystems with or without a wall.

Warming conditions are acting to shift the habitats and behaviours of coastal fish populations. Increasing concentrations of coastal ocean nutrients from agricultural runoff lead to unsightly and potentially toxic harmful algal blooms that ultimately sap coastal waters of oxygen. Elevated temperature and altered chemical conditions also contribute to the bleaching and destruction of coral reefs, which in their prime state act both as oases of biodiversity and natural barriers that protect coastal systems against storm surges. This all results in lower fish stocks, thus impacting the 10% of the global population that relies on fisheries as their sole source of income and main protein source.

As coastal systems are physically and chemically altered, or simply lost to rising waters, so too is their outsized ability to capture carbon from the atmosphere and sequester it away. Up to 1 million hectares of these systems are destroyed each year, liberating greenhouse gases that ultimately fuel more coastal change^2^. The establishment of Marine Protected Areas are a step towards preserving ecological balance and resources, however as these feedbacks indicate, mitigating isolated impacts of climate change are unlikely to be enough for coasts.

On top of the continuous impacts of sea level rise, ocean warming, and chemical shifts, extreme weather events are also increasing. Extreme weather pummels coasts, causing sudden, yet severe damages. The cost of recovering from these events are staggering, as evidenced by Hurricane Katrina in 2005 and Hurricane Harvey in 2017, which cost roughly $160 billion and $125 billion respectively. The recent projections for the 2020 North Atlantic hurricane season estimate there could be around 20 named storms between June and November—which is among the highest estimates in recent years, and particularly foreboding in the midst of the coronavirus pandemic.

If such changes to coastal environments continue unmitigated, the benefits, appeal and safety of coastal living will significantly diminish. The ability of communities to prepare for and respond to prolonged or sudden coastal impacts is determined by socio-political and economic privilege^3^. Over the short term, infrastructure construction to protect against coastal hazards will be more likely in wealthier communities. In the far future—or after a catastrophic event—those with the means to relocate will flee the coasts, whereas those who cannot due to economic or cultural reasons will have no choice but to remain. The consequences of coastal climate change will most devastatingly impact disadvantaged communities, and for the small island nations that are already suffering the effects of rising seas there is no inland to flee to.

Coasts are depicted as a simple line on a map—a seamless switch from terrestrial to marine—but this belies their social and scientific complexity, and their importance to the Earth system. The research in our Coasts collection showcases the multiple complexities that span the physical and biological aspects of coastal systems, and how they are threatened. Put together, we hope these works can serve as a springboard for multidisciplinary research that helps reverse the tide on the many impacts of coastal climate change.
